# Exploring the Obesity Paradox in All Subtypes of Intracranial Hemorrhage: A Retrospective Cohort Analysis of 13,000 Patients

**DOI:** 10.3390/brainsci14121200

**Published:** 2024-11-28

**Authors:** Helen Ng, Ellen N. Huhulea, Ankita Jain, Michael Fortunato, Galadu Subah, Ariel Sacknovitz, Eris Spirollari, Jon B. Rosenberg, Andrew Bauerschmidt, Stephan A. Mayer, Chirag D. Gandhi, Fawaz Al-Mufti

**Affiliations:** 1 School of Medicine, New York Medical College, Valhalla, NY 10595, USA; 2 Department of Neurosurgery, Westchester Medical Center, Valhalla, NY 10595, USA; 3 Department of Neurology, Westchester Medical Center, Valhalla, NY 10595, USA

**Keywords:** stroke, intracranial hemorrhage, subarachnoid hemorrhage, intracerebral hemorrhage, obesity, obesity paradox

## Abstract

Background/Objectives: Recent studies reveal an “obesity paradox”, suggesting better clinical outcomes after intracranial hemorrhage for obese patients compared to patients with a healthy BMI. While this paradox indicates improved survival rates for obese individuals in stroke cases, it is unknown whether this trend remains true across all forms of intracranial hemorrhage. Therefore, the objective of our study was to investigate the incidence, characteristics, and outcomes of hospitalized obese patients with intracranial hemorrhage. Methods: The National Inpatient Sample (NIS) database was queried for data from 2015 to 2019 to identify adult patients aged 18 years and older with a primary diagnosis of non-traumatic intracranial hemorrhage. Using International Classification of Disease 10th Edition codes, patients were stratified by BMI categories: healthy weight, overweight, class I–II obesity, and class III obesity. The cohorts were examined for demographic characteristics, comorbidities, stroke severity, inpatient complications, interventions, and clinical outcomes, including length of stay (LOS), discharge disposition, and inpatient mortality. Results: Of 41,960 intracranial hemorrhage patients identified, 13,380 (33.0%) also had an obese BMI. Class I–II obese intracranial hemorrhage patients were more likely to be of white race (OR: 1.101, 95% CI: 1.052, 1.152, *p* < 0.001), less likely to be female (OR: 0.773, 95% CI: 0.740, 0.808, *p* < 0.001), and more likely to have diabetes mellitus (OR: 1.545, 95% CI: 1.477, 1.616, *p* < 0.001) and hypertension (OR: 1.828, 95% CI: 1.721, 1.943, *p* < 0.001) in comparison to healthy-weight patients. In a matched cohort analysis adjusting for demographics and severity, intracranial hemorrhage patients with class I–II obesity had a shorter length of stay (LOS) (OR 0.402, 95% CI: 0.118, 0.705, *p* < 0.001), reduced inpatient mortality (OR 0.847, 95% CI: 0.798, 0.898, *p* < 0.001), and more favorable discharge disposition (OR 1.395, 95% CI: 1.321, 1.474, *p* < 0.001) compared to their healthy-weight counterparts. Furthermore, these patients were less likely to require decompressive hemicraniectomy (OR 0.697, 95% CI: 0.593, 0.820, *p* < 0.001). Following an analysis of individual ICH subtypes, obese subarachnoid hemorrhage (SAH) patients demonstrated reduced mortality (OR: 0.692, 95% CI: 0.577–0.831, *p* < 0.001) and LOS (OR: 0.070, 95% CI: 0.466–0.660, *p* = 0.039), with no differences in discharge disposition. Similarly, intracerebral hemorrhage patients demonstrated reduced mortality (OR: 0.891, 95% CI: 0.827–0.959, *p* = 0.002) and LOS (OR: 0.480, 95% CI: 0.216–0.743, *p* < 0.001). Other ICH subtypes showed improved discharge outcomes (OR: 1.504, 95% CI: 1.368–1.654, *p* < 0.001), along with decreased mortality (OR: 0.805, 95% CI: 0.715–0.907, *p* < 0.001) and LOS (OR: −10.313, 95% CI: −3.599 to −2.449, *p* < 0.001). Conclusions: Intracranial hemorrhage patients with class I–II obesity exhibited more favorable clinical outcomes than those who were of a healthy weight or overweight. Despite its association with risk factors contributing to intracranial hemorrhage, class I–II obesity was associated with improved outcomes, lending support to the existence of the obesity paradox in stroke.

## 1. Introduction

Obesity remains a widespread public health concern, with a prevalence of 42.4% in the United States (32,487,284) and 14.0% worldwide (35,584,732). These patients carry a high comorbidity burden and are at increased risk of cerebrovascular events [1]. Despite the common predisposition to believe that comorbid obesity may confer a worse prognosis on stroke patients, recent studies reveal an “obesity paradox”, suggesting more favorable outcomes for obese stroke patients than for those with a healthy BMI. This paradox, first noted in patients with elevated BMI showing decreased stroke risk and improved heart failure survival, is now garnering increased interest and support in cerebrovascular disease [2]. Evidence indicates that obese adult stroke patients have a lower mortality rate than their healthy-weight counterparts [3]. A study on acute ischemic stroke (AIS) found significantly lower mortality rates across stroke severity thresholds in obese patients compared to non-obese patients [4]. Additionally, obese AIS patients treated with thrombectomy demonstrated reduced mortality [4].

One retrospective analysis showed that obesity was associated with decreased inpatient mortality and an increased likelihood of routine discharge after intracranial hemorrhage [5]. Moreover, a recent systematic review and meta-analysis reported that obesity was associated with reduced short-term and long-term mortality in intracranial hemorrhage patients as compared to their non-obese counterparts [6]. However, these reviews focused on a single subtype of intracranial hemorrhage, rather than different subtypes, at a time and are thus limited in scope, precluding any definitive conclusions about the obesity paradox from being drawn as it relates to all forms of stroke.

The incidence of both cerebrovascular disease and obesity has seen a notable rise in recent years, a trend that underscores the importance of investigating whether the obesity paradox applies to obese patients across all subtypes of intracranial hemorrhage. No other studies to date have explored the impact of the obesity paradox on mortality rates across all forms of intracranial hemorrhage among adults. This retrospective cohort study aims to bridge this gap by analyzing all intracranial hemorrhage subtypes, with distinct sub-analyses focusing on the following subtypes of intracranial hemorrhage: SAH, intracerebral hemorrhage (ICH), and other/unspecified forms of intracranial hemorrhage (including non-traumatic subdural and epidural hematomas).

## 2. Materials and Methods

### 2.1. Data Source and Patient Selection

The National (Nationwide) Inpatient Sample (NIS) was developed by the Agency for Health Care Research and Quality as part of the Healthcare Cost and Utilization Program (HCUP). It is the largest publicly available, all-payer, inpatient care database in the United States. It contains information from approximately seven million hospital stays each year. For this study, the NIS was queried for data from 2015 to 2019 on adult patients admitted with a diagnosis of non-traumatic intracranial hemorrhage, identified by the International Classification of Diseases 10th Edition (ICD-10) codes for SAH, ICH, and other and unspecified forms of intracranial hemorrhage (Appendix A). Patients were further stratified by body mass index (BMI): healthy weight (BMI 18.5–24.9), overweight (BMI 25–29.9), class I–II obesity (low risk, BMI 30.0–34.9, and moderate risk, BMI 35.0–39.9, respectively), and class III obesity (high risk, BMI ≥ 40.0). Since the NIS is publicly available and contains no identifiable patient information, approval by an institutional review board was not required for this study.

### 2.2. Data Characteristics and Outcomes Measured

Demographic characteristics, including age, sex, and race, were recorded. Comorbidities, such as hypertension and long-term anticoagulation or antiplatelet therapy, were compared between intracranial hemorrhage patients with and without a class I–II obesity BMI. Acute stroke indices were determined using standard components, such as aphasia, dysphagia, cerebral edema, coma, hemiplegia, herniation, and mechanical ventilation. Hemorrhage severity was ascertained with the National Inpatient Sample Subarachnoid Hemorrhage Severity Score (NIH-SSS). The NIS-SSS is a validated measure of SAH severity and was created to address the limitations of previous measures of severity in the analysis of NIS data sets. Inpatient interventions, including decompressive hemicraniectomy (DHC) and external ventricular drainage (EVD) placement, were also examined. To measure clinical outcomes, we analyzed length of stay (LOS), discharge home, and inpatient mortality.

### 2.3. Statistical Analyses

Standard descriptive statistics and multivariable regression analyses were performed to evaluate associations between obese and non-obese patients and clinical outcomes. Categorical variables were compared using Pearson’s chi-squared test. Continuous variables were evaluated using the independent samples *t*-test. Comparisons of demographics, comorbidities, interventions, and complications were made between class I–II obese intracranial hemorrhage patients and non-class-I–II-obese intracranial hemorrhage patients. Multivariate logistic regression was performed to create a 1:1 propensity score nearest-neighbor-matched cohort using the covariates of age, sex, race, and acute stroke indices. Statistical Product and Service Solutions (SPSS) Statistical Software Version 29 was used for analysis, and statistical significance was set at an alpha of 0.05. Missing data were excluded from analysis. If values of any of the variables were missing, the entire case was excluded from the analysis. When calculating frequencies, missing values were excluded, and percentages were based on the number of non-missing values.

## 3. Results

### 3.1. Baseline Demographics and Comorbidities

Our query yielded 41,960 patients diagnosed with intracranial hemorrhage, of which 13,830 (33.0%) had a class I–II obesity BMI. Class I–II obese intracranial hemorrhage patients were more likely to be of white race (OR: 1.101, 95% CI: 1.052, 1.152, *p* < 0.001), have diabetes mellitus (OR: 1.545, 95% CI: 1.477, 1.616, *p* < 0.001), and have hypertension (OR: 1.828, 95% CI: 1.721, 1.943, *p* < 0.001) in comparison to healthy-weight patients. Conversely, these patients were less likely to be female (OR: 0.773, 95% CI: 0.740, 0.808, *p* < 0.001) and to have chronic obstructive pulmonary disorder (COPD) (OR: 0.361, 95% CI: 0.263, 0.495, *p* < 0.001) than the other groups (Table 1).

### 3.2. Complications

Class I–II obese intracranial hemorrhage patients were less likely to develop pneumonia (OR: 0.824, 95% CI: 0.767, 0.886, *p* < 0.001) and pulmonary embolism (OR: 0.845, 95% CI: 0.735, 0.971, *p* = 0.018), and were less likely to have urinary tract infection (OR: 0.877, 95% CI: 0.825, 0.932, *p* < 0.001), acute kidney injury (OR: 0.826, 95% CI: 0.783, 0.870, *p* < 0.001), sepsis (OR: 0.630, 95% CI: 583, 0.680, *p* < 0.001), and anoxic brain damage (OR: 0.832, 95% CI: 0.698, 0.992, *p* = 0.041) than healthy-weight patients (Table 2).

### 3.3. Stroke Variables and Interventions

Class I–II obese intracranial hemorrhage patients were more likely to have aphasia (OR: 1.091, 95% CI: 1.030, 1.157, *p* = 0.003) but were less likely to have cerebral herniation (OR: 0.807, 95% CI: 0.758, 0.858, *p* < 0.001), paresis/plegia (OR: 0.778, 95% CI: 0.697, 0.869, *p* < 0.001), and stupor (OR: 0.601, 95% CI: 0.380, 0.950, *p* = 0.029) than patients in the other strata (Table 2).

Class I–II obese intracranial hemorrhage patients were more likely to have postoperative complications that would require a gastrostomy (OR: 2.037, 95% CI: 0.992, 4.183, *p* < 0.001) or EVD (OR: 2.411, 95% CI: 1.544, 3.766, *p* < 0.001), and less likely to require DHC (OR: 0.697, 95% CI: 0.593, 0.820, *p* < 0.001) compared to healthy-weight patients (Table 3).

### 3.4. Outcomes: All Intracranial Hemorrhages Combined

In a cohort matched for demographics and intracranial hemorrhage severity, class I–II obesity and all forms of intracranial hemorrhage were associated with a decreased LOS (OR: 0.402, 95% CI: 0.118, 0.705), a decreased likelihood of inpatient mortality (OR: 0.847, 95% CI: 0.798, 0.898, *p* < 0.001), and an increased likelihood of routine discharge home (OR: 1.395, 95% CI: 1.321, 1.474, *p* < 0.001) compared to healthy-weight patients (Table 4).

When controlling for coma, a deep state of unconsciousness, class I–II obesity and all forms of intracranial hemorrhage were associated with a decreased LOS (OR: 0.296, 95% CI: 0.117, 0.431, *p* = 0.003), a decreased likelihood of inpatient mortality (OR: 0.873, 95% CI: 0.782, 0.974, *p* = 0.015), and no significant difference in the likelihood of routine discharge home (OR: 0.961, 95% CI: 0.799, 1.55, *p* = 0.671) compared to healthy-weight patients (Table 5).

### 3.5. Outcomes: Subarachnoid Hemorrhage

In a cohort matched for demographics and intracranial hemorrhage severity, class I–II obesity and SAH were associated with a decreased LOS (OR: 0.070, 95% CI: 0.446, 0.660, *p* = 0.039), a decreased likelihood of inpatient mortality (OR: 0.692, 95% CI: 0.577, 0.831, *p* < 0.001), and no significant difference in the likelihood of routine discharge home (OR: 0.829, 95% CI: 0.644, 1.069, *p* = 0.149) compared to healthy-weight patients (Table 6).

When controlling for coma with the Glascow Coma Score, class I–II obesity and SAH were associated with a decreased LOS (OR: 0.791, 95% CI: 0.583, 0.939, *p* = 0.005), a decreased likelihood of in-patient mortality (OR: 0.549, 95% CI: 0.406, 0.742, *p* < 0.001), and no significant difference in the likelihood of routine discharge home (OR: 0.879, 95% CI: 0.526, 1.470, *p* = 0.624) compared to healthy-weight patients (Table 7).

### 3.6. Outcomes: Intracerebral Hemorrhage

In a cohort matched for demographics and intracranial hemorrhage severity, obesity class I and II intracranial hemorrhage patients had a shorter LOS (OR: 0.480, 95% CI: 0.216, 0.743, *p* < 0.001) and a decreased likelihood of inpatient mortality (OR: 0.891, 95% CI: 0.827, 0.959, *p* = 0.002) but no significant difference in the likelihood of routine discharge home (OR: 1.058, 95% CI: 0.980, 1.143, *p* = 0.148) compared to healthy-weight patients (Table 8).

When controlling for coma, obese intracranial hemorrhage patients had a decreased likelihood of inpatient mortality (OR: 0.862, 95% CI: 0.758, 0.981, *p* = 0.024) but no significant difference in LOS (OR: 1.013, 95% CI: 0.010, 1.078, *p* = 0.070) or the likelihood of routine discharge home (OR: 0.871, 95% CI: 0.691, 1.098, *p* = 0.242) compared to healthy-weight patients (Table 9).

### 3.7. Outcomes: Other and Unspecified Forms of Intracranial Hemorrhage

In a cohort matched for demographics and intracranial hemorrhage severity, obesity class I–II patients had a shorter LOS (OR: 0.384, 95% CI: 0.360, 0.449, *p* < 0.001), a decreased likelihood of inpatient mortality (OR: 0.805, 95% CI: 0.715, 0.907, *p* < 0.001), and an increased likelihood of routine discharge home (OR: 1.504, 95% CI: 1.368, 1.654, *p* < 0.001) compared to healthy-weight patients (Table 10).

When controlling for coma, obesity class I and II patients had an increased likelihood of routine discharge home (OR: 2.007, 95% CI: 1.326, 3.038, *p* < 0.001) but no significant difference in LOS (OR: 0.416, 95% CI: 0.116, 1.083, *p* = 0.677) or the likelihood of inpatient mortality (OR: 0.856, 95% CI: 0.670, 1.092, *p* = 0.211) compared to healthy-weight patients (Table 11).

## 4. Discussion

This multicenter, retrospective, nationwide analysis of 41,960 patients with intracranial hemorrhage examined all subtypes of intracranial hemorrhage in adult patients with class I–II obesity compared to adult intracranial hemorrhage patients who were of a healthy weight or overweight. The results reveal improved clinical outcomes, most notably a decreased likelihood of mortality. Intracranial hemorrhage patients with class I–II obesity were less likely to undergo DHC, potentially contributing to the observed mortality benefit. DHC is a surgical intervention that directly relieves the potentially fatal elevation in intracranial pressure following ICH [7,8]. While DHC may improve short-term survival after SAH/ICH, it is also linked to poor functional outcomes and reduced quality of life [9]. These findings suggest an underlying protective factor that may reduce the need for invasive procedures in class I–II obese ICH patients and ultimately result in reduced mortality and adverse outcomes.

The results from our study align with the findings from a previous retrospective analysis, which suggest similarly improved clinical and survival outcomes for class I–II obese patients who experience an intracranial hemorrhage [5]. One possible explanation for this phenomenon is that individuals with obesity BMIs have greater central adiposity, which causes reduced venous return and results in an increased ICP and a decreased cerebral perfusion pressure (CPP) at baseline [10,11,12,13]. Thus, obese patients with intracranial hemorrhage may be better able to tolerate the elevated ICP and decreased CPP associated with acute ischemia in intracranial hemorrhage.

Our findings demonstrated a lower risk of mortality for all intracranial hemorrhages combined, as well as for SAH and ICH. This is in contrast to a study of 406 patients following SAH, which discovered a protective effect in overweight patients, while obese patients exhibited increased mortality and poor outcomes [14]. The most recent NIS study on this subject indicated poorer outcomes in class I–II obese stroke patients who were comatose [5]. After controlling for coma, our multivariate analysis demonstrated a decreased likelihood of inpatient mortality in patients who experienced ICH or SAH, alongside a decreased LOS in cases of SAH, revealing obesity as a consistent protective factor in intracranial hemorrhage outcomes. The observed reduction in inpatient mortality and LOS emphasizes the potential beneficial role of obesity in this patient population, indicating areas for future research and consideration in treatment options for improved patient outcomes.

Although this emerging body of work lends credence to the obesity paradox in stroke, the existence of this phenomenon is still controversial. Our results stand in contrast to those of previous studies that have not observed similar mortality benefits of higher BMI after controlling for severity. For example, a recent study suggested that the paradox may be an artificial finding due to selection bias. In order to avoid this and to control for severity, the authors only examined deaths that occurred within the first 30 days and that were directly caused by the indexed stroke. They found no difference in mortality within the first week and the first month between obese and non-obese patients [15,16]. Another study found that after adjusting for several covariates, including severity, overweight and obesity BMI showed no mortality benefit, while underweight BMI showed increased mortality [17]. Despite the ongoing debate over the obesity paradox, it is critical to continue to study the effect of obesity on adult intracranial hemorrhage patients because of the growing prevalence of obesity and its significant impact on patient care.

The primary limitation of this study is the inherent use of BMI as a metric for assessing obesity. The obesity paradox remains controversial and may be influenced by various confounding factors, such as unintentional weight loss due to illness, different obesity phenotypes, and fitness levels [17,18]. In assuming that excess weight is due to adipose tissue, the BMI scale may incorrectly label healthy individuals with muscular physiques as being overweight or obese. As such, incomplete information comprising measures of obesity could impact the study’s outcomes and prevent a comprehensive understanding of the obesity paradox.

Another limitation is the retrospective nature of the study and the restricted ability to establish an association between treatment modality and clinical outcomes. Interpreting data from a retrospective database presents inherent limitations. As the data were collected for billing purposes rather than for research, it is inevitable that there are missing variables in the database that may have impacted the results if they had been recorded and included. Additionally, multiple outcomes could not be studied simultaneously because retrospective studies rely on identifying the outcome first and then the risk variable, thus limiting the ability to determine cause and effect. However, information gained from this retrospective study may aid in planning for a future prospective study, which may provide more accuracy regarding exposures, outcomes, and confounders and allow for multiple outcomes to be studied at a time. Moreover, one data entry in the NIS corresponds to one hospital admission but does not necessarily correspond to a unique patient. It is possible that a patient with multiple hospitalizations can contribute multiple data entries with different outcomes, which can lead to overestimation of ICH events. Additionally, the NIS is limited in that it reports only 20% of all hospital discharges in the nation and may not accurately capture insurance statuses that can affect data pertinent to outcomes. For example, based on insurance status, some patients may be discharged home without additional services due to socioeconomic status, despite the persistence of disability or other sequelae. However, the sample size and nationally encompassing data promote the generalizability and novelty of the presented findings.

## 5. Conclusions

Our multicenter, retrospective, nationwide analysis bolsters the emerging obesity paradox in stroke by demonstrating that among adult patients suffering from intracranial hemorrhage, those with class I–II obesity BMI exhibit better clinical outcomes. This finding indicates a protective role of obesity in ICH and suggests the importance of considering BMI in treatment decisions for these patients. Specifically, class I–II obesity BMI may influence treatment effectiveness, highlighting the need for tailored interventions. Further research is needed to better understand the mechanisms underlying these observations, including any correlations with venous pathology, and to optimize treatment strategies accordingly.

## Figures and Tables

**Table 1:** Demographics and comorbidities of intracranial hemorrhage patients.

N = 41,960	All ICH Patients	Non-Class I–II Obesity BMI	Obesity BMI (Class I–II)	Univariate OR (95% CI)	Univariate *p*-Value	Multivariate OR (95% CI)	Multivariate *p*-Value
Age	41,960 Mean: 64.67 years SD: 14.630 years	28,130 (67.0%)	13,830 (33.0%)	0.886 (0.880, 0.989)	**<0.001**	0.980 (0.979, 0.982)	**<0.001**
Female	20,360 (48.5%)	14,220 (50.6%)	6140 (44.4%)	0.730 (0.697, 0.784)	**<0.001**	0.773 (0.740, 0.808)	**<0.001**
White	24,470 (58.3%)	16,450 (58.5%)	8020 (58.0%)	1.102 (1.074, 1.154)	**<0.001**	1.101 (1.052, 1.152)	**<0.001**
Diabetes Mellitus	15,560 (37.1%)	9395 (33.4%)	6165 (44.6%)	1.513 (1.477, 1.618)	**<0.001**	1.545 (1.477, 1.616)	**<0.001**
Hypertension	33,525 (79.9%)	21,660 (77.0%)	11,865 (85.8%)	1.829 (1.741, 1.945)	**<0.001**	1.828 (1.721, 1.943)	**<0.001**
Tobacco Use	4675 (11.1%)	3070 (10.9%)	1605 (11.6%)	0.913 (0.901, 1.005)	0.453	0.967 (0.902, 1.035)	0.333
COPD	405 (1.0%)	360 (1.3%)	45 (0.3%)	0.452 (0.262, 0.649)	**<0.001**	0.361 (0.263, 0.495)	**<0.001**

**Table 2:** Complications—all intracranial hemorrhagic strokes.

N = 41,960	All ICH Patients	Non-Class I–II Obesity BMI	Obesity BMI (Class I–II)	Univariate OR (CI 95%)	Univariate *p*-Value	Multivariate OR (CI 95%)	Multivariate *p*-Value
Pneumonia	5520 (13.2%)	4075 (14.5%)	1445 (10.4%)	0.833 (0.791, 0.884)	**<0.001**	0.824 (0.767, 0.886)	**<0.001**
Deep Vein Thrombosis	1690 (4.0%)	1130 (4.0%)	560 (4.0%)	1.081 (0.949, 1.115)	0.257	1.092 (0.972, 1.227)	0.137
Pulmonary Embolism	1220 (2.9%)	870 (3.1%)	350 (2.5%)	0.848 (0.755, 0.974)	**0.019**	0.845 (0.735, 0.971)	**0.018**
Myocardial Infarction	1825 (4.3%)	1240 (4.4%)	585 (4.2%)	1.131 (1.036, 1.261)	**0.029**	1.130 (1.015, 1.259)	**0.026**
Cerebral Edema	13,365 (31.9%)	8790 (31.2%)	4575 (33.1%)	1.033 (0.990, 1.082)	0.214	1.032 (0.983, 1.084)	0.209
Herniation	7485 (17.8%)	5150 (18.3%)	2335 (16.9%)	0.810 (0.756, 0.859)	**<0.001**	0.807 (0.758, 0.858)	**<0.001**
Hydrocephalus	6000 (14.3%)	3915 (13.9%)	2085 (15.1%)	1.315 (0.973, 1.107)	0.358	1.035 (0.963, 1.111)	0.353
Aphasia	7070 (16.8%)	4515 (16.1%)	2555 (18.1%)	1.124 (1.040, 1.158)	**0.006**	1.091 (1.030, 1.157)	**0.003**
Paresis/Plegia	14,260 (34.0%)	1340 (4.8%)	505 (3.7%)	0.782 (0.701, 0.865)	**<0.001**	0.778 (0.697, 0.869)	**<0.001**
Stupor	120 (0.3%)	90 (0.3%)	30 (0.2%)	0.610 (0.394, 0.939)	**0.030**	0.601 (0.380, 0.950)	**0.029**
Atrial Fibrillation	10,175 (24.2%)	6910 (24.6%)	3265 (23.6%)	1.018 (0.941, 1.112)	0.471	1.025 (0.972, 1.081)	0.363
UTI	7310 (17.4%)	5355 (19.0%)	1955 (14.1%)	0.896 (0.844, 0.921)	**<0.001**	0.877 (0.825, 0.932)	**<0.001**
Acute Kidney Injury	10,805 (25.8%)	7625 (27.1%)	3180 (23.0%)	0.853 (0.795, 0.868)	**<0.001**	0.826 (0.783, 0.870)	**<0.001**
Sepsis	5245 (12.5%)	4125 (14.7%)	1120 (8.1%)	0.647 (0.596, 0.670)	**<0.001**	0.630 (0.583, 0.680)	**<0.001**
Anoxic Brain Damage	720 (1.7%)	505 (1.8%)	215 (1.6%)	0.842 (0.703, 0.983)	0.056	0.832 (0.698, 0.992)	**0.041**
Coma	7765 (18.5%)	5115 (18.2%)	2650 (19.2%)	1.297 (0.740, 1.095)	0.815	1.014 (0.946, 1.086)	0.702

**Table 3:** Interventions—all intracranial hemorrhagic strokes.

N = 41,960	All ICH Patients	Non-Class I–II Obesity BMI	Obesity BMI (Class I–II)	Univariate OR (CI 95%)	Univariate *p*-Value	Multivariate OR (CI 95%)	Multivariate *p*-Value
Tracheostomy	2190 (2.8%)	1150 (4.1%)	1040 (7.5%)	0.949 (0.857, 1.319)	0.191	0.912 (0.796, 1.045)	0.184
Gastrostomy	45 (0.1%)	30 (0.1%)	15 (0.1%)	1.607 (0.724, 3.585)	**<0.001**	2.037 (0.992, 4.183)	**<0.001**
Hemicraniotomy (DHC)	695 (0.9%)	350 (1.2%)	345 (2.5%)	0.812 (0.655, 0.711)	**<0.001**	0.697 (0.593, 0.820)	**<0.001**
Intubation	10,515 (13.4%)	5395 (19.2%)	5120 (37.0%)	1.165 (0.783, 1.230)	0.996	1.001 (0.944, 1.062)	0.962
External Ventricular Drainage (EVD)	115 (0.1%)	70 (0.2%)	45 (0.3%)	2.281 (1.379, 3.514)	**<0.001**	2.411 (1.544, 3.766)	**<0.001**

**Table 4:** Intracranial hemorrhagic stroke outcomes stratified by BMI.

N = 41,960	Routine Discharge	In-Hospital Mortality	Length of Stay
Healthy weight	0.550 (0.497, 0.608) ***p* < 0.001**	1.040 (0.951, 1.138) *p* = 0.392	M = 13.89, SD = 18.304 days 6.705 (1.118, 2.042) ***p* < 0.001**
Overweight	1.068 (0.982, 1.162) *p* = 0.125	0.842 (0.770, 0.920) ***p* < 0.001**	M = 13.50, SD = 15.810 days 4.904 (0.682, 1.591) ***p* < 0.001**
Obese (Class I, II)	1.206 (1.138, 1.278) ***p* < 0.001**	0.866 (0.814, 0.921) ***p* < 0.001**	M = 11.29, SD = 13.399 days −12.661 (−2.406, −1.761) ***p* < 0.001**
Obese (Class I–III)	1.351 (1.258, 1.450) ***p* < 0.001**	1.086 (1.012, 1.166) ***p* = 0.023**	M = 12.07, SD = 14.009 days −9.020 (−1.983, −1.275) ***p* < 0.001**
Obese (Class III only)	1.054 (0.988, 1.125) *p* = 0.108	1.259 (1.181, 1.343) ***p* < 0.001**	M = 13.09, SD = 14.683 days 4.687 (0.475, 1.158) ***p* < 0.001**

**Table 5:** Outcomes—all comatose intracranial hemorrhagic stroke patients.

N = 6380	OR (95% CI)	*p*-Value
Discharge Home	0.961 (0.799, 1.155)	0.671
Inpatient Mortality	0.873 (0.782, 0.974)	**0.015**
Mean LOS	0.296 (0.117, 0.431)	**0.003**

**Table 6:** Subarachnoid hemorrhagic stroke outcomes stratified by BMI.

N = 2840	Routine Discharge	In-Hospital Mortality	Length of Stay
Healthy weight	0.525 (0.350, 0.788) ***p* = 0.002**	1.368 (1.051, 1.781) ***p* = 0.020**	M = 16.33, SD = 12.866 days 1.249 (−0.608, 2.741) *p* = 0.212
Overweight	1.049 (0.738, 1.491) *p* = 0.791	0.704 (0.543, 0.914) ***p* = 0.008**	M = 18.28, SD = 20.840 days 3.922 (1.671, 5.012) ***p* < 0.001**
Obese (Class I, II)	0.829 (0.644, 1.069) *p* = 0.149	0.692 (0.577, 0.831) ***p* < 0.001**	M = 14.68, SD = 16.336 days −2.070 (−2.446, −0.066) ***p* = 0.039**
Obese (Class I–III)	1.324 (0.988, 1.774) *p* = 0.060	0.992 (0.809, 1.216) *p* = 0.938	M = 14.72, SD = 15.262 days −3.770 (−3.796, −1.198) ***p* < 0.001**
Obese (Class III only)	1.685 (1.275, 2.227) ***p* < 0.001**	1.479 (1.219, 1.794) ***p* < 0.001**	M = 14.77, SD = 13.711 days −1.460 (−2.212, 0.324) *p* = 0.144

**Table 7:** Outcomes—comatose subarachnoid hemorrhagic stroke.

N = 965	OR (95% CI)	*p*-Value
Discharge Home	0.879 (0.526, 1.470)	0.624
Inpatient Mortality	0.549 (0.406, 0.742)	**<0.001**
Mean LOS	0.791 (0.583, 0.939)	**0.005**

**Table 8:** Intracerebral hemorrhagic stroke outcomes stratified by BMI.

N = 21,650	Routine Discharge	In-Hospital Mortality	Length of Stay (95% CI)
Healthy weight	0.493 (0.423, 0.574) ***p* < 0.001**	0.863 (0.765, 0.975) ***p* = 0.018**	M = 14.62, SD = 17.771 days 5.714 (1.218, 2.491) ***p* < 0.001**
Overweight	1.260 (1.126, 1.409) ***p* < 0.001**	0.904 (0.807, 1.011) *p* = 0.078	M = 13.64, SD = 14.284 days 2.510 (0.168, 1.369) ***p* = 0.012**
Obese (Class I, II)	1.058 (0.980, 1.143) *p* = 0.148	0.891 (0.827, 0.959) ***p* = 0.002**	M = 11.96, SD = 14.806 days −8.480 (−2.157, −1.347) ***p* < 0.001**
Obese (Class I–III)	1.211 (1.100, 1.334) ***p* < 0.001**	1.155 (1.055, 1.264) ***p* = 0.002**	M = 12.61, SD 14,709 days −6.270 (−1.972, −1.033) ***p* < 0.001**
Obese (Class III only)	1.092 (1.006, 1.186) ***p* = 0.036**	2.129 (1.642, 2.760) ***p* < 0.001**	M = 13.43, SD = 14.530 days 3.424 (0.277, 1.124) ***p* = 0.001**

**Table 9:** Outcomes—comatose intracerebral hemorrhagic stroke.

N = 4580	OR (95% CI)	*p*-Value
Discharge Home	0.871 (0.691, 1.098)	0.242
In-patient Mortality	0.862 (0.758, 0.981)	**0.024**
Mean LOS	1.013 (0.010, 1.078)	**0.070**

**Table 10:** Outcomes—other forms of intracranial hemorrhagic stroke.

N = 11,510 OR (95% CI) *p*-Value	Routine Discharge	In-Hospital Mortality	Length of Stay
Healthy Weight	0.599 (0.521, 0.689) ***p* < 0.001**	1.362 (1.181, 1.572) ***p* < 0.001**	M = 13.84, SD = 21.755 days 7.324 (1.966, 3.403) ***p* < 0.001**
Overweight	0.825 (0.722, 0.941) ***p* = 0.004**	0.755 (0.648, 0.881) ***p* < 0.001**	M = 12.64, SD = 16.809 days 3.115 (0.438, 1.927) ***p* = 0.002**
Obese (Class I, II)	1.504 (1.368, 1.654) ***p* < 0.001**	0.805 (0.715, 0.907) ***p* < 0.001**	M = 9.76, SD = 9.644 days −10.313 (−3.599, −2.449) ***p* < 0.001**
Obese (Class I–III)	1.623 (1.454, 1.811) ***p* < 0.001**	0.956 (0.846, 1.081) *p* = 0.472	M = 10.76, SD = 12.254 days −8.532 (−3.119, −1.954) ***p* < 0.001**
Obese (Class III only)	0.978 (0.876, 1.092) *p* = 0.694	1.192 (1.055, 1.348) ***p* = 0.005**	M = 12.14, SD = 14.995 days 2.084 (0.040, 1.292) ***p* = 0.037**

**Table 11:** Outcomes—comatose patients with other forms of intracranial hemorrhagic stroke.

N = 1465	OR (95% CI)	*p*-Value
Routine Discharge	2.007 (1.326, 3.038)	**<0.001**
Inpatient Mortality	0.856 (0.670, 1.092)	0.211
Mean LOS	0.416 (0.116, 1.083)	0.677

## Data Availability

The study data can be requested from the corresponding author after completing the required procedures outlined by the Healthcare Cost and Utilization Project.

## Appendix A

**Table A1:** Diagnosis codes.

Diagnosis	International Classification of Diseases, 10th Revision, Clinical Modification and Procedure Codes
Healthy BMI	Z681–Z684
Overweight BMI	Z6825–Z6829
Class I–II obesity BMI	Z683
Class III obesity BMI	Z684, Z685
Subarachnoid hemorrhage	I60
Intracerebral hemorrhage	I61
Other and unspecified hemorrhage	I62
Diabetes mellitus	E10, E11
Hypertension	I10–I13
Tobacco use	F1721
COPD	J41–J43
Pneumonia	J12–J18
Pulmonary embolism	I26
Myocardial infarction	I21
Cerebral edema	G936
Herniation	G935
Hydrocephalus	G91
Aphasia	R47
Paresis/plegia	R295, I6933–I6936
Stupor	R401
Atrial fibrillation	I48
Urinary tract infection	N39
Sepsis	A41
Anoxic brain damage	G931
Acute kidney injury	N17

Diagnosis

International Classification of Diseases, 10th Revision, Clinical Modification and Procedure Codes

**Table A2:** Diagnosis codes.

Procedure	International Classification of Diseases, 10th Revision, Clinical Modification and Procedure Codes
Tracheostomy	0B11, 0B110, 0B110D, 0B110D6, 0B110F, 0B110F4, 0B110Z, 0B110Z4, 0B113, 0B113F, 0B113F4, 0B113Z, 0B113Z4, 0B114, 0B114F, 0B114F4, 0B114Z, 0B114Z4
Gastrostomy	0BH6, 0BH60, 0BH60G, 0BH60GZ, 0BH63, 0BH63G, 0BH63GZ, 0BH64, 0BH64G, 0BH64GZ, 0BH67, 0BH67G, 0BH67GZ, 0BH68, 0BH68G, 0BH68GZ, 0D16, 0D160, 0D1607, 0D16074, 0D16079, 0D1607A, 0D1607B, 0D1607L, 0D160J4, 0D160J9, 0D160JA, 0D160JB, 0D160JL, 0D160K, 0D160K4, 0D160K9, 0D160KA, 0D160KB, 0D160KL, 0D160Z4, 0D160Z9, 0D160ZA, 0D160ZB, 0D160ZL, 0D163, 0D163J, 0D163J4, 0D164, 0D1647, 0D16474, 0D16479, 0D1647A, 0D1647B, 0D1647L, 0D164J, 0D164J4, 0D164J9, 0D164JA, 0D164JB, 0D164JL, 0D164K, 0D164K4, 0D164K9, 0D164KA, 0D164KB, 0D164KL, 0D164Z, 0D164Z4, 0D164Z9, 0D164ZA, 0D164ZB, 0D164ZL, 0D168, 0D1687, 0D16874, 0D16879, 0D1687A, 0D1687B, 0D1687L, 0D168J, 0D168J4, 0D168J9, 0D168JA, 0D168JB, 0D168JL, 0D168K, 0D168K4, 0D168K9, 0D168KA, 0D168KB, 0D168KL, 0D168Z, 0D168Z4, 0D168Z9, 0D168ZA, 0D168ZB, 0D168ZL
Hemicraniotomy	00J00ZZ, 0N800ZZ, 0W9100Z, 0W910ZZ, 0WC10ZZ, 0N500ZZ, 0NB00ZZ, 0NT10ZZ, 0NT30ZZ, 0NT40ZZ, 0NT50ZZ, 0NT60ZZ, 0NT70ZZ, 009000Z, 00900ZZ, 00C00ZZ, 00B70ZZ, 00500ZZ, 00B00ZZ, 00T70ZZ
Intubation	0BH13EZ, 0BH17EZ, 0BH18EZ, 5A1935Z, 5A1945Z, 5A1955Z
External ventricular drainage	00760ZZ, 00763ZZ, 00764ZZ, 00B60ZX, 00B60ZZ, 00B63XZ, 00B63ZZ, 00B64XZ, 00B64ZZ, 00W60ZZ, 00W602Z, 00W603Z, 00W60JZ, 00W60MZ, 00W60YZ, 00W630Z, 00W632Z, 00W633Z, 00W63JZ, 00W63MZ, 00W63YZ, 00W640Z, 00W642Z, 00W643Z, 00W64JZ, 00W64MZ, 00W64YZ, 00W6X0Z, 00W6X2Z, 00W6X3Z, 00W6XJZ, 00W6XMZ, 03LM0DZ, 03LM0ZZ, 03LM3BZ, 03LM3CZ, 03LM3DZ, 03LM3ZZ, 03LM4BZ, 03LM4CZ, 03LM4DZ, 03LM4ZZ
